# Order policy for emergency medicine with return uncertainty in a closed-loop supply chain

**DOI:** 10.1371/journal.pone.0205643

**Published:** 2018-10-25

**Authors:** Wei Pan, Ying Guo, Wenji Zhang, Lei Jin, Shujie Liao

**Affiliations:** 1 School of Economic and Management, Wuhan University, Wuhan, China; 2 School of Management, Shandong University, Jinan, China; 3 Cancer Biology Research Center, Tongji Hospital, Tongji Medical College, Huazhong University of Science and Technology, Wuhan, Hubei, PR China; Florida State University, UNITED STATES

## Abstract

Due to difficulties in accurately predicting the emergency timing and the magnitude of a disaster, operations for perishable emergency inventory planning often encounter expiration and shortage problems. In order to ease the expiration problem in emergency medicine preparation inventories, this paper investigates an emergency medicine closed-loop supply chain for returning unused items from an ERC (Emergency Reserve Center) to a hospital. To assure that the return strategy is meaningful, we propose a critical parameter that we term the latest return time, after which the remaining emergency medicine in the ERC cannot be returned to the hospital. In addition, the short lifetime of emergency products and uncertainty about demand time and demand quantity are also considered in this emergency inventory planning system. In analyzing the optimal ordering policies, we find that the two threshold values for the predefined return time, which affect the total costs, are not monotonous; rather, the direction of their effect is first down, then up, and then down again, which means that a better predefined value of the latest return time can be determined by minimizing total costs. By studying and comparing decentralized and centralized decisions, we find that the centralized decision system works better to control expiration and costs. Therefore, we design a coordination mechanism for the cooperation between the ERC and the hospital. Our analysis shows that we should not ignore the emergency uncertainty and perishability of emergency items.

## 1. Introduction

To prepare for man-made or natural disasters, many cities maintain an emergency reserve center (referred to hereafter as ERC) to store key emergency items, like food, water, and medicine. In this paper, we focus on emergency medicine, which is important and indispensable. The emergency medicines typically stored in an ERC include anti-flu drugs and vaccines, which are time-sensitive and have short lifetimes. In China, investment in emergency supplies is significant, because of the many serious disasters that occur. This investment causes financial concern related to the problems of medicine expiration and shortage, and the expense of storage (Meng et al., 2017) [1]. As a consequence of this, senior managers go to great lengths to implement strategies that balance the need for high fulfillment levels of demand from affected populations with the need to minimize costs. Furthermore, although the lifetime of some kinds of emergency items may be as long as several years (e.g., gloves and first aid kits), the probability of a disaster is low enough that most emergency items in the ERC expire before being used, particularly products that are time-sensitive and have short lifetimes (Guide et al., 2006) [2]. Given these difficulties and the significance of emergencies, it is imperative to propose ways in which emergency medical supplies can be managed. Analyzing how to deal with the expiration problem in the emergency inventory planning system, and how to contain the expiration problem using the proposed closed-loop emergency supply chain, is necessary and significant.

Management must also overcome limited product disposition options, which can lead to substantial losses in product value recovery. Referring to the achievement of Zhou and Olsen (2017) [3], and observing hospitals’ regular demand, we propose a closed-loop supply chain that includes the ERC, the hospital and the supplier. In our system, both the ERC and the hospital order from the supplier at the beginning of the period. Then, the ERC sends its remaining stock to the hospital before it expires at the predefined latest return time or at the end of the emergency response period, and the ERC replenishes its emergency inventory with new items at the same time. There are two questions we should examine: why the hospital should receive the unused medicines from the ERC, and when the ERC should adopt the closed-loop supply chain strategy. The first question can be solved easily, since the holding value of the emergency medicine decays as time passes, and the hospital pays less money for the unused medicines, which have the same value as compared with new medicines from suppliers. The second question is more complicated, and in this paper, we seek to establish a balance between return costs and return benefits, in order to effectively reduce waste.

Given our efforts to reduce the expiration problem for short lifetime emergency supplies with uncertainties about occurrence time and the closed-loop emergency supply chain, we develop quantitative models to answer the following research questions:

1. What are the effects of introducing a closed-loop strategy for the emergency inventory planning system? Moreover, does the closed-loop strategy reduce both the expiration losses and the shortage losses experienced by a particular emergency inventory system during a disaster?
2. To the best of our knowledge, the emergency preparation process is constrained by uncertain occurrence time and perishability. Therefore, what are the impacts of stochastic occurrence time and emergency medicine perishability?
3. What are the differences between decentralized and centralized decisions on the optimal emergency inventory level of the ERC and the ordering quantity of the hospital?

To answer these research questions, we develop models under newsboy settings using a closed-loop supply chain for a hospital, an ERC and a supplier for perishable emergency supplies. The two distinctive features of these kinds of perishable emergency supplies are: (i) limited warehousing time, and (ii) uncertainties in demand time and demand quantity. Motivated by insights about uncertainties on occurrence time and demand quantity in a perishable emergency supplies inventory system, our proposed return process is seen as uncertainty, because in this system, the return quantity is equal to the remaining quantity after an emergency response, and the return time is equal to the ending time of the emergency response. Therefore, the closed-loop supply chain for emergency medicines proposed in this paper is different from other traditional inventory systems in its basic assumptions, and it is also different from the rotation system proposed in the work of Zhou and Olsen (2017) [3] for an uncertain return process.

Derived from the emergency medicine inventory system, this paper tries to make sense of operational insights for emergency medicine ordering policies of ERCs and hospitals. The operational insight we note here includes specifically how to implement an effective inventory system for a closed-loop emergency medicine supply chain and when to use this closed-loop strategy. To better understand the practical operations of an emergency inventory system, we analyze equilibrium solutions that balance risk costs and reserve costs. Risk costs include shortage costs, while reserve costs include expiration costs, return costs and storage costs. Thus, this paper makes three contributions. First, this study extends the scope of the existing literature by synthesizing prior research issues, namely perishable inventory control and emergency management. Second, this article offers a closed-loop perishable emergency supply chain strategy derived from the uncertainties about occurrence time and severity of damage. Third, our study is combined with both the decentralized and centralized decision models and illustrates an integrated, efficient perishable emergency inventory system.

The reminder of this paper is organized as follows. Section 2 reviews the relevant literature pertaining to perishability return and the emergency medicine inventory system. Section 3 presents assumptions about the emergency medicine supply chain abstracted from real world situation to simplify research problems, and builds inventory models for the ERC and the hospital. Section 4 characterizes and discusses optimal ordering policies. Section 5 develops a centralized model for the ERC and the hospital, and compares the results with those of a decentralized system. Section 6 conducts numerical case studies to intuitively test our results and describe the sensitivity of key factors on the optimal policies. Finally, relevant conclusions and implications are drawn in Section 6.

## 2. Literature review

Research on inventory systems is quite comprehensive; therefore, extending this work becomes a matter of intensive work on a specific aspect; our work focuses on the topics of an emergency item inventory system, a perishable item inventory system and an inventory return system.

The emergency inventory system has received considerable attention from researchers, and Ozguven and Ozbay (2014) [4] provided a comprehensive review on emergency inventory management for disasters. Most studies focus on operating issues: the pre-positioning, distribution and scheduling of the reserved emergency resources prepared for an emergency response (Duran et al., 2011; Anaya-Arenas et al., 2014; Ruth and Erhan, 2016) [5–7]. In terms of emergency situations, the process is stochastic. Stochastic demand, capacity, lead-time, price, effectiveness and cost are the most common stochastic variables in optimization problems (Louly et al., 2008; Charles, 2012) [8–9]. However, in this article, we consider the occurrence time and demand as stochastic variables, following the example of Pan et al. (2015) [10] and Meng et al. (2017) [1] did. Pan et al. (2015) [10] considered the risks of expiration and items being out of stock because of stochastic occurrence time and demand, and, using CVaR (conditional value at risk), they studied how decision-makers managed emergency inventories with different risk attitudes. Meng et al. (2017) [1] studied how to control expiration and costs for emergency inventory management. In our study, we complement the efforts of these earlier studies by incorporating the uncertainty or randomness of occurrence time, as well as limited warehousing times in emergency preparations, using a stochastic mathematical model derived from commonly used newsboy theories (Kabak and Schi, 1978; Gallego and Moon, 1993) [11–12].

Since we also consider short lifetime emergency products, we briefly review perishable inventory studies. Nahmias (1975) [13] and Fries (1975) [14] contend with the short lifetime problem and both conclude that the product age distribution affects optimal inventory polices. To our knowledge, few papers study the perishable problem for emergency medical resources inventory management. Shen, Dessouky, and Ordonez (2011) [15] proposed a modified EMQ model for studying the expiration problem in the national medical reserve with a minimum volume constraint. However, the many different kinds of emergency products and different age distributions for each product create complexity, and later researchers have concluded approximate equilibriums that do not consider age distribution (Zhou and Olsen, 2017) [3]. Some researchers went on to analyze the perishable inventory system by simplifying age distribution by looking at a unique inventory product or at only one period (Pan et al., 2015; Meng et al., 2017) [10, 1]. In our emergency inventory system, given that many emergency materials, such as alcohol, blood and plasma have short life cycles (Guo et al., 2018) [16], it is imperative that efforts are made to improve emergency product inventory management processes by combining a return strategy with a stochastic programming approach.

Referring to the return strategy, most prior research on this issue of a closed-loop return supply chain concentrates on commercial products (Wang et al., 2007; Subulan et al., 2015) [17–18], particularly those with short life cycles (Xu at al., 2015; Li at al., 2016) [19–20]. In this perspective, Hasani et al. (2012) [21] proposed a stochastic model that deals with the issues of limited warehousing times and closed-loop supply chain network designs for the high-tech and food industries. Fortunately, in an emergency management system, Meng et al. (2017) [1] might be the first study to adopt two replacement mechanisms (based on remaining lifetime and remaining quantity) for dealing with the problem of emergency material expiration. Zhou and Olsen (2017) [3] proposed a rotation system between a national medical reserve and a hospital to solve the expiration problem in the emergency inventory system. An assessment of these studies shows that a successful replacement strategy can help to reduce the costs and risks level associated with the management process. On the basis of these prior achievements, we propose an emergency medicine closed-loop supply chain in which the return process is uncertain, especially in emergency situations.

A more secure and efficient inventory management system for disasters can be built by integrating emergency smart technologies, such as Ratio Frequency Identification Devices (RFIDs), for commodity tracking and logistics; such devices can also be used for an online emergency inventory control system [22–25]. This paper makes a unique contribution by studying the emergency inventory plan, which should be decided before a disaster strikes, rather than focusing on emergency inventory control during a disaster response. We further involve smart technologies in our emergency inventory planning systems, which can utilize real data and incorporate the transformation laws of disasters to develop more efficient and scientific emergency inventory systems. An analysis of the literature about offline inventory management shows that having an emergency inventory system is an important issue, especially for addressing the perishable problem and the return strategy design. However, little empirical effort has been used to examine the emergency medicine closed-loop supply chain and the effect of the stochastic return process on optimal ordering policies. Our study builds on the efforts of existing studies by introducing a stochastic return process that utilizes the newsboy approach to effectively control expiration and shortage problems. The main contribution of our work is proposing optimal policies for the ERC and the hospital in decentralized and centralized settings when facing a stochastic return process.

## 3. Assumptions and models

### 3.1. Assumptions

We consider the ordering problem from the view of single period in a short lifetime product inventory system with an Emergency Reserve Center (ERC), a hospital and a supplier. The ERC orders only from the supplier, and keeps certain emergency medicines to prepare for an emergency response but does not stock products that are in regular (non-emergency) demand. When the demand for an emergency response is lower than the stock levels, the remaining emergency medicines are returned to the hospital at the end of an emergency response time *t*; when there is no demand, all emergency medicines in the ERC are returned to the hospital before the expiry date at a predefined time *θT*, and the ERC immediately replenishes inventory up to the decided stock level.

The hospital receives emergency items from the supplier and the ERC; however, the received quantity and time of receipt is uncertain. That is to say, the process of returning the stock to the hospital is uncertain, because of the stochastic occurrences of time and demand. The return quantity equals the remaining quantity after an emergency response, and the return time equals the ending time of the emergency response. This assumption means that both the return quantity and return time are stochastic. The received time from the ERC should occur before the expiry date, which we call the latest return time *θT* where 0 ≤ *θ* ≤ 1; it is not meaningful to return expired items that have no value.

**Fig 1:**
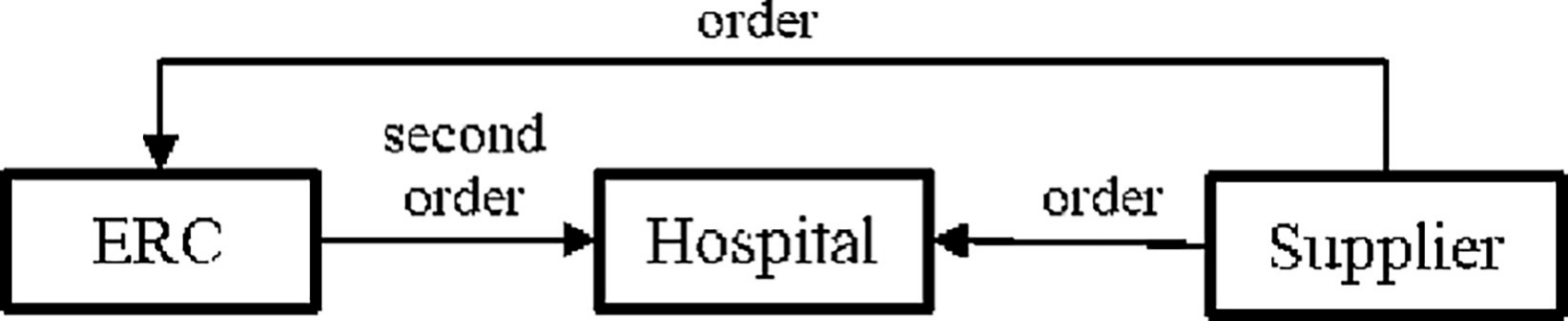
Emergency medicine supply chain.

Both the ERC and the hospital order from the supplier, and all the orders eventually go to the supplier. The inventory returned by the ERC to the hospital is replenished by the ERC from the supplier, so that the ERC maintains its predetermined optimal stock level. The closed-loop emergency medicine supply chain is presented as Fig 1. Decisions include: how much emergency medicine the ERC should order from the supplier, i.e., *I*, and how much emergency medicine the hospital should order from the supplier, i.e., *Q*. In this proposed return system, the interaction of the hospital with the ERC is uncertain, which affects the optimal ordering decisions for both the ERC and the hospital.

We assume that the ERC and the hospital are decentralized and that decisions are made by different decision-makers. Thus, the hospital needs to pay for the returns, even though the price of emergency medicine is linear, declining as time passes and the medicines age (approach their expiry date). The ERC should take on costs if transferring inventory (e.g., transportation costs), and receive the salvage revenue of returns at the same time. All unsatisfied demand at the ERC and hospital cause shortage losses, while items in the hospital’s inventory that go that expire at the end of the single period incur expiration costs. Therefore, the total costs of the ERC can be classified as shortage cost, transport cost, holding cost, and salvage revenue; and the total costs of the hospital include ordering cost, shortage cost, expiration cost, and holding cost.

The hospital faces regular demand for emergency medicine with demand rate *y*. Two scenarios are considered: scenario 1 is $\theta T\leq \frac{Q}{y}$, and scenario 2 is $\theta T>\frac{Q}{y}$. We develop the models under both scenarios in Section 3.2, and we discuss the case of scenario 2, which is less tractable when deriving optimal ordering policies. In addition to this difficulty, the hospital will respond to a more serious shortage risk in scenario 2; therefore, the case of scenario 2 is not as rigid in the emergency medicine inventory system.

The emergency response is quick, which is feasible, because the time value function requires a quicker response for a disaster (Meng et al., 2017) [1]. Considering this characteristic of an emergency response, we assume that the return of unused emergency medicine occurs at the time that a disaster occurs *t*. We assume that one or no disaster occurs within the single short lifetime of a given medicine, because the likelihood of two or more disasters occurring in such a short time interval is low (Pan et al., 2015) [10]. The case of many disasters happening across a longer period is discussed in Section 7, in which one shelf life horizon can be divided into multiple periods based on the occurrence time of disasters.

In the first stage, the ERC decides how many emergency materials should be ordered from the supplier. In the second stage, the hospital decides how much emergency medicine to be ordered from the supplier given return uncertainty.

For the purpose of our analysis, the following notations are used:

*e*: Expired cost per emergency medicine for the hospital

*s*_1_: Shortage cost per emergency medicine for the ERC

*s*_2_: Shortage cost per emergency medicine for the hospital

*h*_1_: Holding cost per time per emergency medicine for the ERC

*h*_2_: Holding cost per time per emergency medicine for the hospital

*r*: Transportation cost from ERC to hospital per emergency medicine

*θ*: Lifetime ratio of emergency medicine for the second purchase by the hospital from the ERC, 0 ≤ *θ* ≤ 1

*T*: Shelf life of emergency products

*y*: Demand rate of hospital for emergency medicines

*x*: Stochastic demand of emergency medicines at stage of emergency response

*t*: Stochastic civil aviation occurrence time

*a*,*b*: The lower and upper bounds of stochastic occurrence time *t*

*c*,*d*: The lower and upper bounds of stochastic demand *x*

*f*(*x*): Probability density function of stochastic demand

*g*(*t*): Probability density function of stochastic occurrence time

*F*(*x*): Cumulative density function of stochastic demand

*G*(*t*): Cumulative density function of stochastic occurrence time

*I*: The optimal inventory level of ERC, a decision variable

*Q*: The optimal order quantity of hospital from supplier, a decision variable

*v*_*t*_: The value of emergency medicine at time *t*, which is linear and declines as *v*_*t*_ = *v* − *kt*. *v* is the initial value of the emergency medicine and *k* is the declining rate.

*Q* ≤ *yT* and *Q* + *I* ≥ *yT*, both of which are reasonable, because if *Q* > *yT*, then the hospital always pay for out-of-date and waste losses; if *Q* + *I* < *yT*, then the hospital always pay for shortage costs.

In emergency practice, comparing the shortage loss and the value of emergency medicine, it is more reasonable to assume that *s*_1_ ≥ *s*_2_ ≥ *v* ≥ *e*.

### 3.2. Models

Scenario 1. $\theta T\leq \frac{Q}{y}$, for the hospital, no shortage would occur before *θT*, which is the latest time for the medicine to reach the hospital in the return phase.

**Fig 2:**
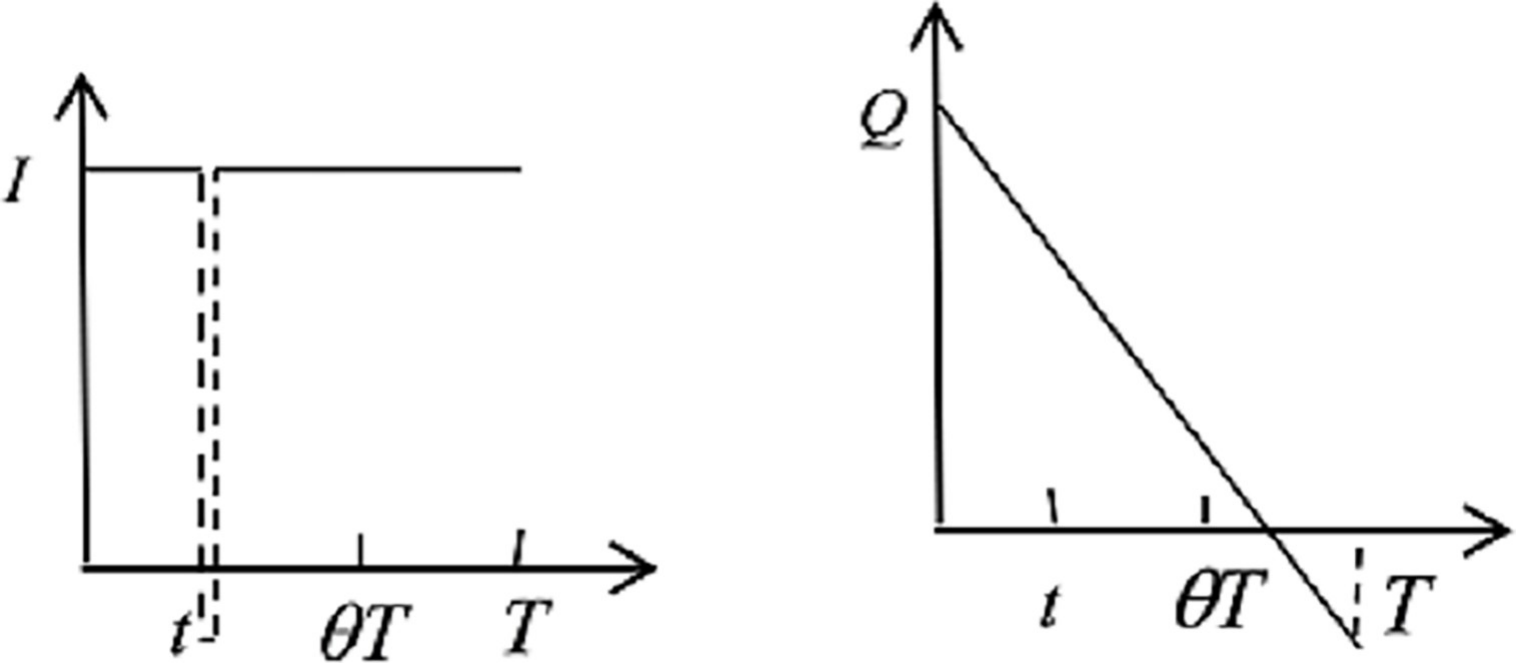
Emergency medicine inventories of ERC and hospital.

1) When a disaster happens within the *θT*, and the demand is larger than the optimal inventory level: the return strategy cannot be conducted by the ERC and the hospital, and the emergency medicine inventories for the ERC and the hospital change, as shown in Fig 2.

$$\mathrm{For}\;\mathrm{ERC}:\;\int_{a}^{\theta T}\int_{I}^{d}\;s_{1}(x-I)f(x)g(t)dxdt+\int_{a}^{\theta T}\int_{I}^{d}\;h_{1}I\;Tf(x)g(t)dxdt$$ (1)

The first term of Eq (1) is the shortage costs and the second term is the holding costs.

$$\mathrm{For}\;\mathrm{hospital}:\;\int_{a}^{\theta T}\int_{I}^{d}\;s_{2}(yT-Q)f(x)g(t)dxdt+\int_{a}^{\theta T}\int_{I}^{d}\;h_{2}\frac{Q}{2}\frac{Q}{y}f(x)g(t)dxdt$$ (2)

Similarly, the first term of Eq (2) is the shortage losses and the second term is the holding costs.

**Fig 3:**
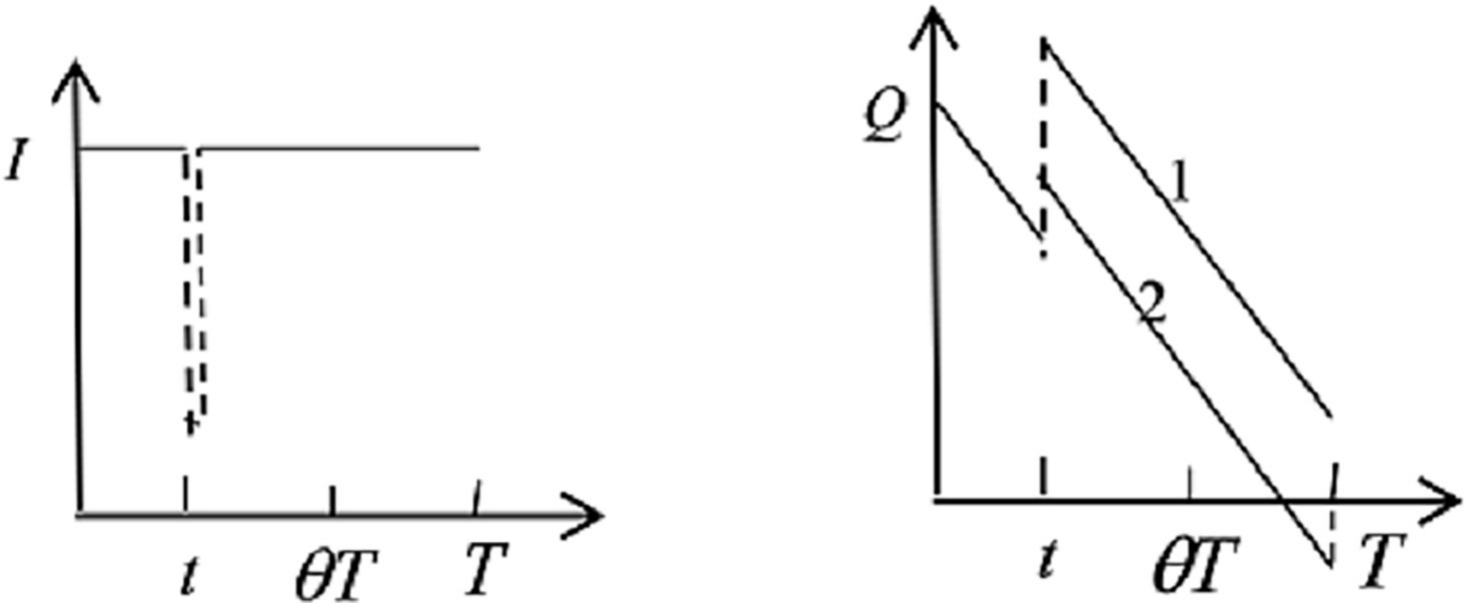
Emergency medicine inventories of ERC and hospital.

2) When a disaster happens within the *θT*, and the ERC has unused inventory, the hospital would receive supplements from the ERC because of the return strategy. These two medicine inventories changes are presented as Fig 3.

For ERC:

$$\begin{array}{l} -\int_{a}^{\theta T}\int_{c}^{I}\;v_{t}(I-x)f(x)g(t)dxdt+\int_{a}^{\theta T}\int_{c}^{I}\;r(I-x)f(x)g(t)dxdt+\\ \int_{a}^{\theta T}\int_{c}^{I}\;h_{1}I\;Tf(x)g(t)dxdt \end{array}$$ (3)

Eq (3) expresses the salvage revenue, the transportation costs and the holding costs.

For hospital:

$$\begin{array}{l} \int_{a}^{\theta T}\int_{c}^{I}v_{t}(I-x)f(x)g(t)dxdt+\int_{a}^{\theta T}\int_{I+Q-yT}^{I}s_{2}(yT-(Q+I-x))f(x)g(t)dxdt+\\ \int_{a}^{\theta T}\int_{c}^{I+Q-yT}h_{2}((Q+I-x)T-\frac{yT}{2}T-(I-x)t)f(x)g(t)dxdt+\\ \int_{a}^{\theta T}\int_{I+Q-yT}^{I}h_{2}(\frac{(Q+Q-yt)t}{2}+\frac{{\left(Q-yt+I-x\right)}^{2}}{2y})f(x)g(t)dxdt+\\ \int_{a}^{\theta T}\int_{c}^{I+Q-yT}e(Q+I-x-yT)f(x)g(t)dxdt \end{array}$$ (4)

The costs expression of Eq (4) includes ordering costs, shortage losses, expiration losses and holding costs of the two cases.

**Fig 4:**
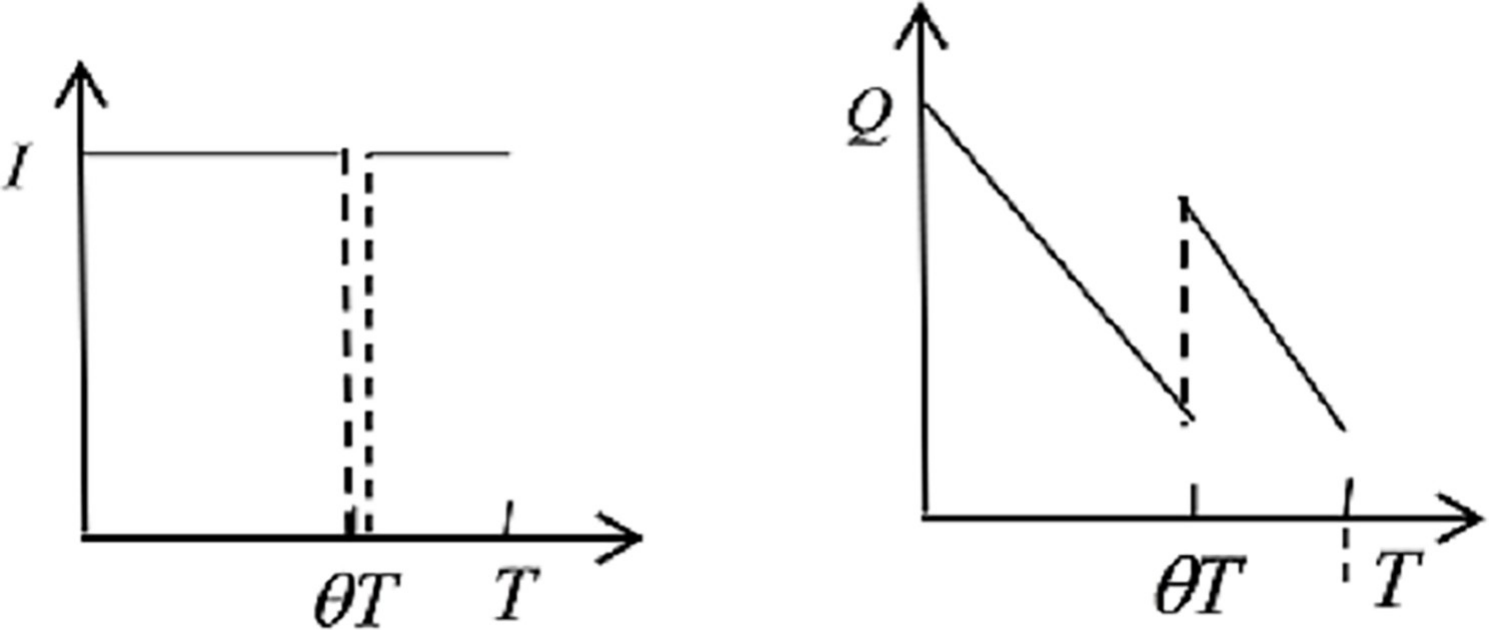
Emergency medicine inventories of ERC and hospital.

3) When no disaster happens within the *θT*, the hospital receives *I* emergency medicines from the ERC in the return process. See Fig 4 to understand the medicine inventories variations intuitively.

$$\mathrm{For}\;\mathrm{ERC}:\;-\int_{\theta T}^{b}v_{\theta T}I\;g(t)dt+\int_{\theta T}^{b}rI\;g(t)dt+\int_{\theta T}^{b}h_{1}I\;Tg(t)dt$$ (5)

The above Eq (5) consists of salvage revenue, transportation costs and holding costs.

For hospital:

$$\int_{\theta T}^{b}v_{\theta T}I\;g(t)dt+\int_{\theta T}^{b}e(I+Q-yT)g(t)dt+\int_{\theta T}^{b}h_{2}((I+Q)T-\frac{yT^{2}}{2}-It)g(t)dt$$ (6)

The inventory costs of the hospital in Eq (6) include ordering costs, expiration losses and holding costs.

Therefore, the total expected costs of the ERC are:

$$\begin{array}{l} C_{ERC}=\int_{a}^{\theta T}\int_{I}^{d}s_{1}(x-I)f(x)g(t)dxdt-\int_{a}^{\theta T}\int_{c}^{I}v_{t}(I-x)f(x)g(t)dxdt-\\ \int_{\theta T}^{b}v_{\theta T}I\;g(t)dt+\int_{a}^{\theta T}\int_{c}^{I}\;r(I-x)f(x)g(t)dxdt+\int_{\theta T}^{b}rI\;g(t)dt+h_{1}I\;T \end{array}$$ (7)

And the total expected costs of the hospital are:

$$\begin{array}{l} C_{H}=\int_{a}^{\theta T}\int_{I}^{d}s_{2}(yT-Q)f(x)g(t)dxdt+\int_{a}^{\theta T}\int_{I}^{d}h_{2}\frac{Q}{2}\frac{Q}{y}f(x)g(t)dxdt+\\ \int_{a}^{\theta T}\int_{c}^{I}v_{t}(I-x)f(x)g(t)dxdt+\int_{a}^{\theta T}\int_{I+Q-yT}^{I}s_{2}(yT-(Q+I-x))f(x)g(t)dxdt\\ +\int_{a}^{\theta T}\int_{c}^{I+Q-yT}e(Q+I-x-yT)f(x)g(t)dxdt+\int_{\theta T}^{b}e(I+Q-yT)g(t)dt+\\ \int_{a}^{\theta T}\int_{c}^{I+Q-yT}h_{2}((Q+I-x)T-\frac{yT}{2}T-(I-x)t)f(x)g(t)dxdt+\\ \int_{\theta T}^{b}h_{2}((I+Q)T-\frac{yT^{2}}{2}-It)g(t)dt+\int_{\theta T}^{b}v_{\theta T}I\;g(t)dt \end{array}$$ (8)

Scenario 2. $\theta T>\frac{Q}{y}$, and the hospital may respond to shortage losses within the *θT* (e.g., the latest time for emergency medicines to reach the hospital in the return phase), because both the occurrence time and demand caused by the disaster are uncertain, resulting in the supplement time and quantity being stochastic. In addition, this situation is the same as scenario 1 for the ERC. Therefore, the ERC analyses are omitted in this scenario.

When a disaster happens within the *θT*, and the demand is higher than the optimal inventory level, the return strategy cannot be operated by the ERC and the hospital.

For hospital:

$$\begin{array}{l} \int_{a}^{Q/y}\int_{I}^{d}s_{2}(yT-Q)f(x)g(t)dxdt+\int_{Q/y}^{\theta T}\int_{I}^{d}s_{2}(yT-Q)f(x)g(t)dxdt+\\ \int_{a}^{Q/y}\int_{I}^{d}h_{2}\frac{Q}{2}\frac{Q}{y}f(x)g(t)dxdt+\int_{Q/y}^{\theta T}\int_{I}^{d}h_{2}\frac{Q}{2}\frac{Q}{y}f(x)g(t)dxdt \end{array}$$ (9)

**Fig 5:**
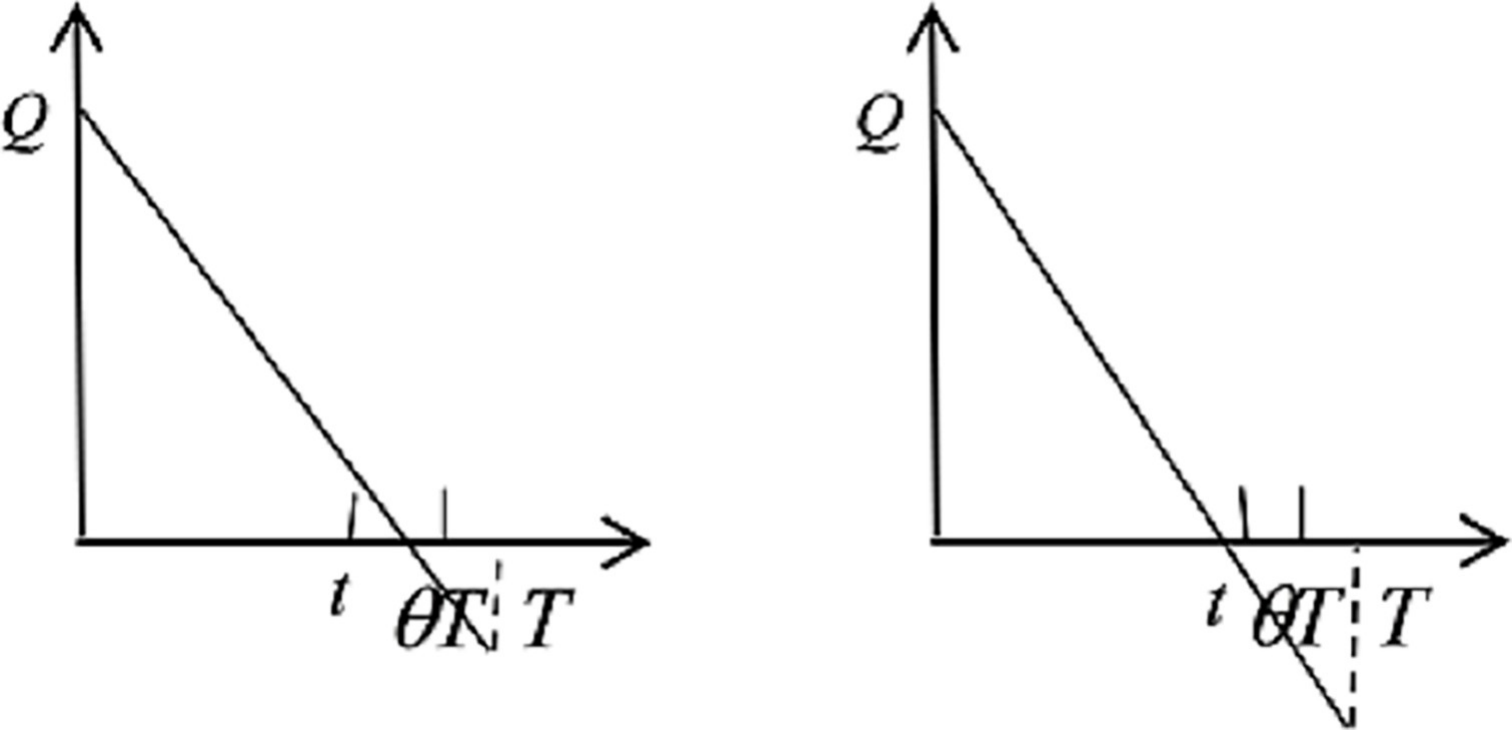
Emergency medicine inventory of hospital for two cases.

The first and second terms of Eq (9) are the shortage losses in the two cases, as shown in Fig 5. The third and fourth terms are the holding costs in the same two cases.

When a disaster happens within the *θT*, and inventory remains, the hospital would receive supplements from the ERC because of the return strategy.

For hospital:

$$\begin{array}{l} \int_{a}^{\theta T}\int_{c}^{I}v_{t}(I-x)f(x)g(t)dxdt+\int_{a}^{Q/y}\int_{I+Q-yT}^{I}s_{2}(yT-(Q+I-x))f(x)g(t)dxdt+\\ \int_{Q/y}^{\theta T}\int_{c}^{I-y(T-t)}s_{2}(yt-Q)f(x)g(t)dxdt+\int_{a}^{Q/y}\int_{c}^{I+Q-yT}e(Q+I-x-yT)f(x)g(t)dxdt\\ +\int_{Q/y}^{\theta T}\int_{I-y(T-t)}^{I}s_{2}((yt-Q)+y(T-t)-(I-x))f(x)g(t)dxdt+\\ \int_{Q/y}^{\theta T}\int_{I-y(T-t)}^{I}e(I-x-y(T-t))f(x)g(t)dxdt+\\ \int_{a}^{Q/y}\int_{c}^{I+Q-yT}h_{2}((Q+I-x)T-\frac{yT}{2}T-(I-x)t)f(x)g(t)dxdt+\\ \int_{a}^{Q/y}\int_{I+Q-yT}^{I}h_{2}(\frac{(Q+Q-yt)t}{2}+\frac{{\left(Q-yt+I-x\right)}^{2}}{2y})f(x)g(t)dxdt+\\ \int_{Q/y}^{\theta T}\int_{c}^{I-y(T-t)}h_{2}(\frac{Q}{2}\frac{Q}{y}+\frac{\left(I-x+I-x-y(T-t))(T-t\right)}{2})f(x)g(t)dxdt+\\ \int_{Q/y}^{\theta T}\int_{I-y(T-t)}^{I}h_{2}(\frac{Q}{2}\frac{Q}{y}+\frac{I-x}{2}\frac{I-x}{y})f(x)g(t)dxdt \end{array}$$ (10)

**Fig 6:**
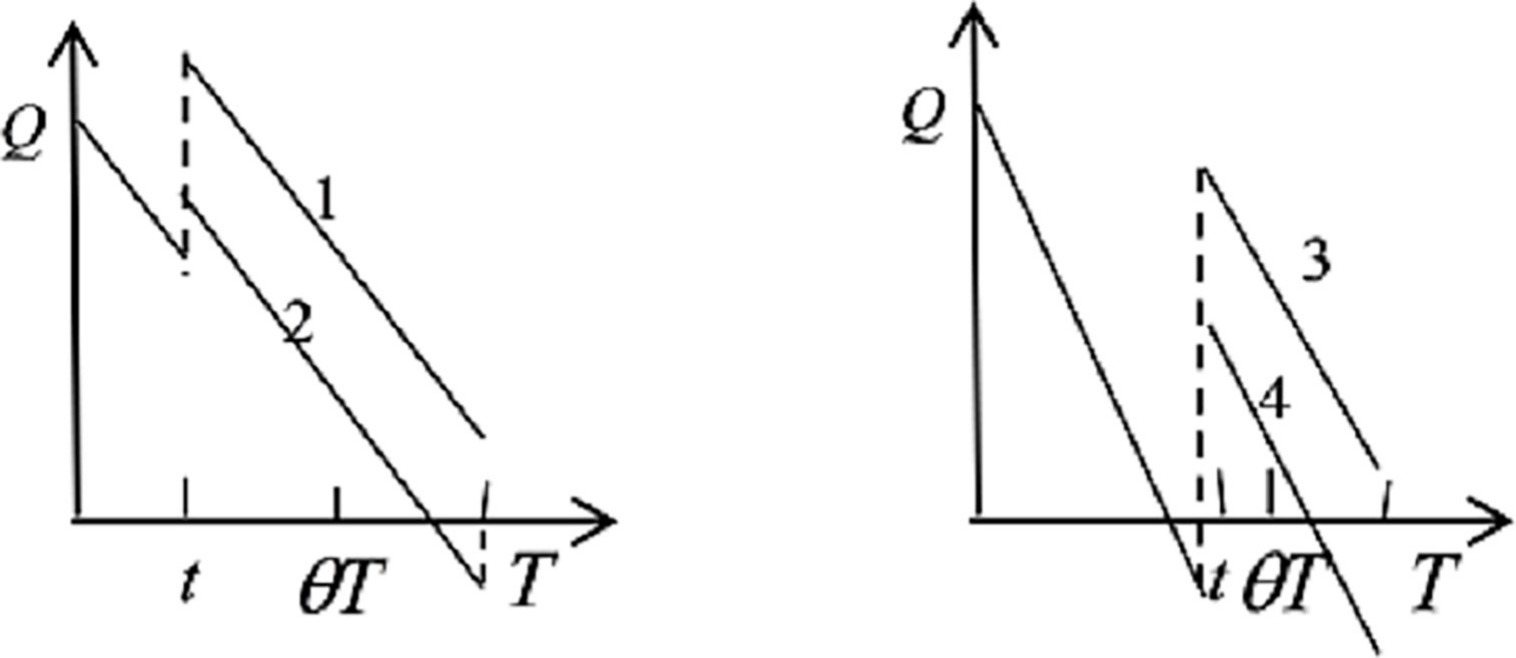
Emergency medicine inventory of hospital for four cases.

This expression of Eq (10) is complicated for the four possible situations. Thus, the first term is the ordering costs, and the second to fourth terms are the expected shortage losses in situations 2, 3, 4, as presented in Fig 6. Similarly, the fifth and sixth terms are expiration losses in situations 1 and 3. The last four terms express the holding costs, representing situations 1, 2, 3 and 4, respectively.

When no disaster happens within the *θT*, the hospital receives *I* emergency medicines from the ERC in the return process.

For hospital:

$$\begin{array}{l} \int_{\theta T}^{b}v_{\theta T}I\;g(t)dt+\int_{\theta T}^{b}s_{2}(y\theta T-Q)g(t)dt+\int_{\theta T}^{b}e(I-y(T-t))g(t)dt+\\ \int_{\theta T}^{b}h_{2}(\frac{Q}{2}\frac{Q}{y}+\frac{\left(I+I-y(T-\theta T))(T-\theta T\right)}{2})g(t)dt \end{array}$$ (11)

**Fig 7:**
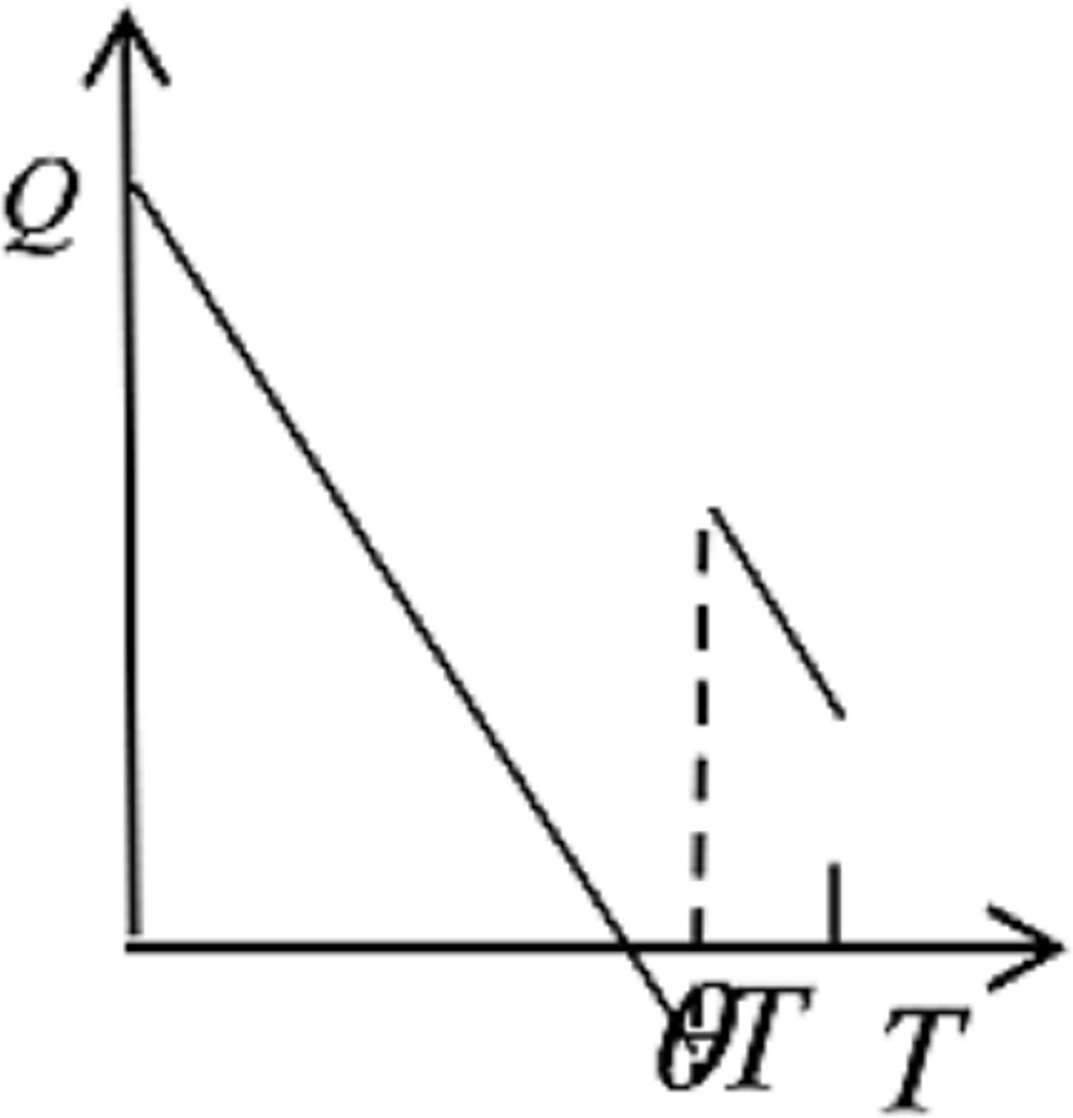
Emergency medicine inventory of hospital for one case.

As shown in Fig 7, the hospital takes on the ordering costs and shortage losses within the *θT*, the expiration losses at the end of the shelf life period, and the holding costs.

Therefore, the total expected costs of ERC, which is the same with Eq (7):

$$\begin{array}{l} C_{ERC}=\int_{a}^{\theta T}\int_{I}^{d}s_{1}(x-I)f(x)g(t)dxdt-\int_{a}^{\theta T}\int_{c}^{I}v_{t}(I-x)f(x)g(t)dxdt-\int_{\theta T}^{b}v_{\theta T}I\;g(t)dt\\ +\int_{a}^{\theta T}\int_{c}^{I}r(I-x)f(x)g(t)dxdt+\int_{\theta T}^{b}rI\;g(t)dt+h_{1}I\;T \end{array}$$

And the total expected costs of hospital:

$$\begin{array}{l} C_{H}=\int_{a}^{\theta T}\int_{I}^{d}s_{2}(yT-Q)f(x)g(t)dxdt+\int_{a}^{\theta T}\int_{I}^{d}h_{2}\frac{Q}{2}\frac{Q}{y}f(x)g(t)dxdt+\\ \int_{a}^{\theta T}\int_{c}^{I}v_{t}(I-x)f(x)g(t)dxdt+\int_{a}^{Q/y}\int_{I+Q-yT}^{I}s_{2}(yT-(Q+I-x))f(x)g(t)dxdt+\\ \int_{Q/y}^{\theta T}\int_{c}^{I}s_{2}(yt-Q)f(x)g(t)dxdt+\int_{Q/y}^{\theta T}\int_{I-y(T-t)}^{I}s_{2}(y(T-t)-(I-x))f(x)g(t)dxdt\\ +\int_{\theta T}^{b}s_{2}(y\theta T-Q)g(t)dt+\int_{a}^{Q/y}\int_{c}^{I+Q-yT}e(Q+I-x-yT)f(x)g(t)dxdt+\\ \int_{Q/y}^{\theta T}\int_{I-y(T-t)}^{I}e(I-x-y(T-t))f(x)g(t)dxdt+\int_{\theta T}^{b}e(I-y(T-t))g(t)dt+\\ \int_{\theta T}^{b}v_{\theta T}Ig(t)dt+\int_{a}^{Q/y}\int_{c}^{I+Q-yT}h_{2}((Q+I-x)T-\frac{yT}{2}T-(I-x)t)f(x)g(t)dxdt+\\ \int_{a}^{Q/y}\int_{I+Q-yT}^{I}h_{2}(\frac{(Q+Q-yt)t}{2}+\frac{{\left(Q-yt+I-x\right)}^{2}}{2y})f(x)g(t)dxdt+\\ \int_{Q/y}^{\theta T}\int_{c}^{I-y(T-t)}h_{2}(\frac{Q}{2}\frac{Q}{y}+\frac{\left(I-x+I-x-y(T-t))(T-t\right)}{2})f(x)g(t)dxdt+\\ \int_{\theta T}^{b}h_{2}(\frac{Q}{2}\frac{Q}{y}+\frac{\left(I+I-y(T-\theta T))(T-\theta T\right)}{2})g(t)dt \end{array}$$ (12)

## 4. The optimal policies

This section derives optimal solutions for the models built in section 3. The process begin by solving for the optimal order quantity Q* for the hospital, and then searching for the optimal inventory level I* of the ERC, which is contrary to the decision sequence, such that this method is a hysteron-protoron scheme.

In Scenario 2 where *Q* ≤ *yθT*, it is found that the optimal solutions are affected by the distributed functions of stochastic demand and the occurrence time is affected more seriously than in Scenario 1. The above finding is intuitive, because the likelihood that the hospital may run out of stock within the *θT* depends on the stochastic demand and occurrence time. That is to say, the hospital would take on more shortage losses, even though it would reduce holding costs. However, comparing the shortage losses and the holding costs in emergency situations, the hospital would take the shortage problem more seriously. In addition to this, governments usually require the ERC to maintain a high minimum stock level, so that it may complete a higher target of demand fulfillment for the affected population after a disaster occurs.

Therefore, in the following analysis, we only consider the situation in which *Q* ≥ *yθT*; that is, we confine the feasible interval to *yθT* ≤ *Q* ≤ *yT* for the ordering quantity of the hospital. Some characteristics of the optimal ordering policies in Scenario 2 are presented and explained in our numerical simulations.

First, we calculate the optimal policy of the hospital for the optimal ordering quantity from the supplier, considering the known information of ERC inventory level *I*.

$$\begin{array}{l} \frac{dC_{H}}{dQ}=-\int_{a}^{\theta T}\int_{I+Q-yT}^{d}s_{2}f(x)g(t)dxdt+\int_{a}^{\theta T}\int_{I}^{d}h_{2}Q/yf(x)g(t)dxdt+\\ \int_{a}^{\theta T}\int_{c}^{I+Q-yT}ef(x)g(t)dxdt+\int_{a}^{\theta T}\int_{I+Q-yT}^{I}h_{2}\left(Q+I-x\right)/yf(x)g(t)dxdt\\ +\int_{a}^{\theta T}\int_{c}^{I+Q-yT}h_{2}Tf(x)g(t)dxdt+\int_{\theta T}^{b}eg(t)dt+\int_{\theta T}^{b}h_{2}Tg(t)dt \end{array}$$ (13)

$$\begin{array}{l} \frac{d^{2}C_{H}}{dQ^{2}}=\int_{a}^{\theta T}s_{2}f(I+Q-yT)g(t)dt+\int_{a}^{\theta T}ef(I+Q-yT)g(t)dt\\ \;+\int_{a}^{\theta T}\int_{I+Q-yT}^{d}h_{2}/yf(x)g(t)dxdt \end{array}$$ (14)

**Fig 8:**
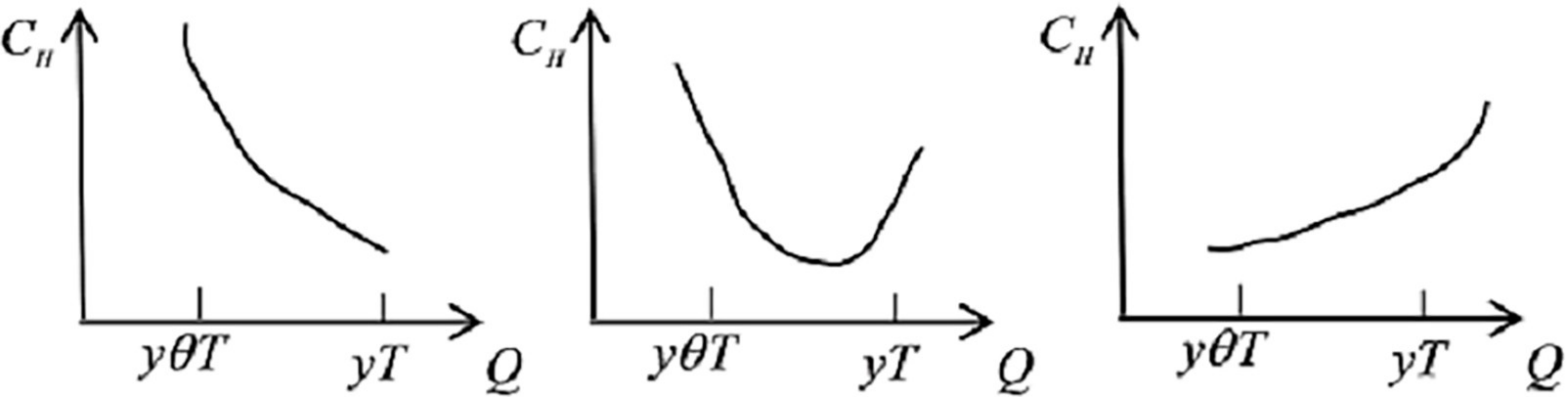
The trends of hospital costs with the order quantity from supplier.

From Eq (14), it is obvious that *d*^2^*C*_*H*_/*dQ*^2^ ≥ 0, which illustrates that the unique optimal solution for the hospital’s ordering policy from the supplier exists if *yT* ≥ Q* ≥ *yθT*. Therefore, the unique optimal solution can be derived by the decreasing or increasing trend of *C*_*H*_ with *Q*, as shown in Fig 8.

For simplicity, Eq (13) is expanded by Eq (15).

$$\begin{array}{l} \frac{dC_{H}}{dQ}=e(1-G(\theta T))+h_{2}T(1-G(\theta T))+s_{2}(F(I+Q-yT)-1)G(\theta T)\\ +h_{2}\frac{Q}{y}G(\theta T)(1+\int_{I+Q-yT}^{I}F(x)dx)+eF(I+Q-yT)G(\theta T) \end{array}$$ (15)

**Proposition 1**. Confining *Q* ≥ *θyT*, the optimal ordering quantity for hospital is:

1. If *eF*(*I*)*G*(*θT*) + *e*(1 − *G*(*θT*)) + *h*_2_*T* ≤ *s*_2_(1 − *F*(*I*))*G*(*θT*), then Q* = *yT*.
2. If $s_{2}(1-F(I-(1-\theta )yT))G(\theta T)\leq h_{2}T(1-G(\theta T))+h_{2}\theta TG(\theta T)+e(1-G(\theta T))+eF(I-(1-\theta )yT)G(\theta T)+G(\theta T)h_{2}/y\int_{I-(1-\theta )yT}^{I}F(x)dx$, then Q* = *θyT*.
3. If the both of above two conditions ((1)-(2)) are not satisfied, the Q* could be derived from the following equations:
  $$\begin{array}{l} s_{2}(1-F(I+Q^{*}-yT))G(\theta T)=e(1-G(\theta T))+eF(I+Q^{*}-yT)G(\theta T)\\ +h_{2}T(1-G(\theta T))+h_{2}Q^{*}/yG(\theta T)+h_{2}/yG(\theta T)\int_{I+Q^{*}-yT}^{I}F(x)dx \end{array}$$

The statements in Proposition 1 are intuitive, and the proofs follow the above process of solving and analysis. The optimal ordering quantity must balance the shortage cost, expiration cost and holding cost, and the optimal solution is not affected by the ordering cost of the returns.

The first statement suggests that the system order *yT* to meet all demand in the hospital, because the expected shortage losses are so large that no shortage is admitted. As indicated by the first statement, the marginal shortage cost is larger than the marginal costs of expiration and holding at the near interval of *yT*. However, reducing the order quantity from the supplier would increase the likelihood that the hospital may run out of stock and decrease the possibility that the emergency medicines may expire. Thus, reducing one unit of order quantity from *yT* will increase the total costs of hospital by *s*_2_(1 − *F*(*I*))*G*(*θT*) − (*eF*(*I*)*G*(*θT*) + *e*(1 − *G*(*θT*)) + *h*_2_*T*). In contrast, according to the expression shown in the second statement, the marginal costs of expiration and holding are larger than the marginal shortage cost at the near interval of *θyT*. Increasing the order quantity from the supplier would decrease the likelihood that the hospital may run out of stock and increase the possibility that the emergency medicines may expire. Thus, enhancing one unit of order quantity from *θyT* will increase the total costs. Therefore, in this second situation, the optimal ordering quantity of the hospital from the supplier is *θyT*. Similar analysis is done for the third statement, and the results show that enhancing one unit of order quantity from *θyT* will decrease the total costs of hospital, and reducing one unit of order quantity from *yT* will decrease the total costs. In the third situation, the optimal ordering quantity of the hospital is Q*, as expressed in the third statement.

Second, we study the optimal policy of ordering quantity for the ERC from the supplier, given the known parameters, including the latest time for return *θ*.

$$\begin{array}{l} \frac{dC_{ERC}}{dI}=-s_{1}(1-F(I))G(\theta T)-(v-k\theta T)F(I)G(\theta T)-kF(I)\int_{a}^{\theta T}G(t)dt\\ +rF(I)G(\theta T)-(v-\theta T)(1-G(\theta T))+r(1-G(\theta T))+h_{1}T \end{array}$$ (16)

$$\frac{d^{2}C_{ERC}}{dI^{2}}=(s_{1}-v+k\theta T+r)f(I)G(\theta T)-kf(I)\int_{a}^{\theta T}G(t)dt$$ (17)

From Eq (17), which refers to the mean value theorem of integrals, it can be understood that *d*^2^*C*_*ERC*_/*dI*^2^ ≥ (*s*_1_ − *v* + *ka* + *r*)*f*(*I*)*G*(*θT*) ≥ 0 based on reasonable assumptions. Therefore, for the ERC, there is a unique optimal inventory policy I*, which can be derived from Eq (16):

$$F(I^{*})=\max(0,\min(\frac{s_{1}G(\theta T)-(v-k\theta T)G(\theta T)+rG(\theta T)+(v-k\theta T)-r-h_{1}T}{s_{1}G(\theta T)-(v-k\theta T)G(\theta T)+rG(\theta T)-k\int_{a}^{\theta T}G(t)dt},1))$$

**Proposition 2**. The optimal inventory policy of the ERC

(1) When $(v-k\theta T)-r-h_{1}T\geq -k\int_{a}^{\theta T}G(t)dt$, then I* = *b*. (2) When (*s*_1_ − (*v* − *kθT*) + *r*)*G*(*θT*) + *v* – *kθT* − *r* − *h*_1_*T* ≤ 0 then I* = *a*.
(3) When $-(s_{1}-(v-k\theta T)+r)G(\theta T)<(v-k\theta T)-r-h_{1}T<-k\int_{a}^{\theta T}G(t)dt$, then $I^{*}=F^{-1}(\frac{s_{1}G(\theta T)-(v-k\theta T)G(\theta T)+rG(\theta T)+(v-k\theta T)-r-h_{1}T}{s_{1}G(\theta T)-(v-k\theta T)G(\theta T)+rG(\theta T)-k\int_{a}^{\theta T}G(t)dt})$.

The ordering decision depends on the size of the parameters, and it is clear that the optimal ordering decision seeks the balance between risk level and costs; a lower risk level brings higher costs and vice versa. Because $0\leq (s_{1}-v+ka+r)G(\theta T)\leq (s_{1}-v+k\theta T+r)G(\theta T)-k\int_{a}^{\theta T}G(t)dt$, the analysis only considers the situation of whether the term of the numerator is positive or negative. In addition, $(s_{1}-(v-k\theta T)+r)G(\theta T)\geq k\int_{a}^{\theta T}G(t)dt$, demonstrating that the third statement is feasible and possible.

The first statement illustrates that the ERC should order *b* units of emergency medicine from the supplier. Simplifying the expression in the first statement, we obtain $(v-k\theta T)+k\int_{a}^{\theta T}G(t)dt\geq r+h_{1}T$; that is, the marginal net expected benefit from the return salvage is larger than the cost of transportation and inventory management. Thus, choosing a higher stock level can increase the salvage revenue. Contrarily, if the marginal shortage cost is less than the costs of value decay, transferring and inventory management, choosing the minimum stock level *a* is a viable option. Otherwise, an equilibrium must be found to balance shortage losses, transferring costs, inventory management costs and salvage revenue, as described in the third statement.

Third, by substituting the parameter *I* in the expression of Q* with I*, we obtain the optimal ordering quantity for the hospital from the supplier as expressed by known and fixed parameters.

Simplify, we define $\Delta =\frac{s_{1}G(\theta T)-(v-k\theta T)G(\theta T)+rG(\theta T)+(v-k\theta T)-r-h_{1}T}{s_{1}G(\theta T)-(v-k\theta T)G(\theta T)+rG(\theta T)-k\int_{a}^{\theta T}G(t)dt}$, $\begin{array}{l} \Phi (I)=\frac{dC_{H}}{dQ}|{}_{Q=\theta yT}=h_{2}T(1-G(\theta T))+h_{2}\theta TG(\theta T)+G(\theta T)h_{2}/y\int_{I-(1-\theta )yT}^{I}F(x)dx\\ \;+e(1-G(\theta T))+eF(I-(1-\theta )yT)G(\theta T)-s_{2}(1-F(I-(1-\theta )yT))G(\theta T) \end{array}$, and $\Psi (I)=\frac{dC_{H}}{dQ}|{}_{Q=yT}=eF(I)G(\theta T)+e(1-G(\theta T))+h_{2}T-s_{2}(1-F(I))G(\theta T)$ in the following analysis. In addition, we demonstrate that *d*Φ(*I*)/*dI* ≥ 0 and *d*Ψ(*I*)/*dI* ≥ 0.

**Theorem 1**. Since *Q* is confined to *θyT* ≤ *Q* ≤ *yT*, the optimal stock policies for the ERC and the hospital by decision sequence are:

1. If Δ ≥ 1, then I* = *b*. Thus, Q* = *θyT* when Φ(*b*) ≥ 0, and Q* ∈ (*θyT*,*yT*) as shown in following when Φ(*b*) ≤ 0.
  $$\begin{array}{l} s_{2}(1-F(b+Q^{*}-yT))G(\theta T)=h_{2}T(1-G(\theta T))+h_{2}Q^{*}/yG(\theta T)\\ +h_{2}/yG(\theta T)\int_{b+Q^{*}-yT}^{b}F(x)dx+e(1-G(\theta T))+eF(b+Q^{*}-yT)G(\theta T) \end{array}$$
2. If Δ ≤ 0, then I* = *a*. So, Q* = *θyT* when Φ(*a*) ≥ 0, Q* = *yT* when Ψ(*a*) ≤ 0, and Q* is expressed as following when Φ(*a*) ≤ 0 and Ψ(*a*) ≥ 0.
  $$\begin{array}{l} s_{2}(1-F(a+Q^{*}-yT))G(\theta T)=h_{2}T(1-G(\theta T))+h_{2}Q^{*}/yG(\theta T)\\ +h_{2}/yG(\theta T)\int_{a+Q^{*}-yT}^{a}F(x)dx+e(1-G(\theta T))+eF(a+Q^{*}-yT)G(\theta T) \end{array}$$
3. If 0< Δ < 1, then I* = *F*^−1^(Δ). Therefore, Q* = *yT* when Ψ(*F*^−1^(Δ)) ≤ 0, Q* = *θyT* when Φ(*F*^−1^(Δ)) ≥ 0, and Q* is derived from the following expression when Φ(*F*^−1^(Δ)) ≤ 0 and Ψ(*F*^−1^(Δ)) ≥ 0.
  $$\begin{array}{l} s_{2}(1-F(F^{-1}(\Delta )+Q^{*}-yT))G(\theta T)=h_{2}T(1-G(\theta T))+h_{2}Q^{*}/yG(\theta T)\\ +h_{2}/yG(\theta T)\int_{F^{-1}(\Delta )+Q^{*}-yT}^{F^{-1}(\Delta )}F(x)dx+e(1-G(\theta T))+eF(F^{-1}(\Delta )+Q^{*}-yT)G(\theta T) \end{array}$$

As shown in Theorem 1, there are three cases for the ERC’s ordering policy from the supplier in situations that involve occurrence time and demand uncertainties: ordering for the largest amount of demand, ordering for the minimum amount of demand, and ordering for a balanced amount of demand. The hospital’s optimal ordering policies also involve three cases with a stochastic return quantity and return time: ordering the maximum stock level—the hospital orders all supplies from the supplier to meet all demand; ordering the minimum stock level—the hospital orders the required minimum stock level *θyT* from the supplier and any other (1 − *θ*)*yT* demands are satisfied by the return strategy or are unsatisfied; ordering the equilibrium amount—the hospital orders the decided equilibrium quantity from the supplier to meet some demand (greater than *θyT*) and the remaining demand is met by means of the return policy, or it is lost. However, if the ERC has ordered the largest amount of demanded supplies from the supplier, then the hospital would not order the maximum amount of supplies from the supplier. Since the highest stock level in the ERC means that the ERC has unused emergency medicines after an emergency response, then there must be expired items in the hospital at the end of the period if the hospital orders the maximum amount; thus, it is better for the hospital to seek an equilibrium order amount in all circumstances.

## 5. Centralized decisions

To find the best possible result in this closed-loop supply chain, we develop a decision setting by which the ERC and the hospital are centralized. In the centralized setting, the ERC would not charge the hospital for returns, it would take on the costs of transferring inventory. This centralized setting can be accomplished through some practical contracts, and this problem should be researched in greater depth in future work.

Combining Eqs (7) and (8), we get the following expression:

$$\begin{array}{l} C^{c}=\int_{a}^{\theta T}\int_{I}^{d}s_{1}(x-I)f(x)g(t)dxdt-\int_{a}^{\theta T}\int_{c}^{I}v_{t}(I-x)f(x)g(t)dxdt-\\ \int_{\theta T}^{b}v_{\theta T}I\;g(t)dt+\int_{a}^{\theta T}\int_{c}^{I}r(I-x)f(x)g(t)dxdt+\int_{\theta T}^{b}rI\;g(t)dt+h_{1}I\;T\\ \int_{a}^{\theta T}\int_{I}^{d}s_{2}(yT-Q)f(x)g(t)dxdt+\int_{a}^{\theta T}\int_{I}^{d}h_{2}\frac{Q}{2}\frac{Q}{y}f(x)g(t)dxdt+\\ \int_{a}^{\theta T}\int_{c}^{I}v_{t}(I-x)f(x)g(t)dxdt+\int_{a}^{\theta T}\int_{I+Q-yT}^{I}s_{2}(yT-(Q+I-x))f(x)g(t)dxdt+\\ \int_{a}^{\theta T}\int_{c}^{I+Q-yT}e(Q+I-x-yT)f(x)g(t)dxdt+\int_{\theta T}^{b}e(I+Q-yT)g(t)dt+\\ \int_{a}^{\theta T}\int_{I+Q-yT}^{I}h_{2}(\frac{(Q+Q-yt)t}{2}+\frac{{\left(Q-yt+I-x\right)}^{2}}{2y})f(x)g(t)dxdt+\\ \int_{a}^{\theta T}\int_{c}^{I+Q-yT}h_{2}((Q+I-x)T-\frac{yT}{2}T-(I-x)t)f(x)g(t)dxdt+\\ \int_{\theta T}^{b}h_{2}((I+Q)T-\frac{yT^{2}}{2}-It)g(t)dt+\int_{\theta T}^{b}v_{\theta T}I\;g(t)dt \end{array}$$ (18)

$$\begin{array}{l} \frac{\partial C^{c}}{\partial I}=-s_{1}(1-F(I))G(\theta T)+rF(I)G(\theta T)+r(1-G(\theta T))\\ \;+h_{1}T+e(1-G(\theta T))+h_{2}T-h_{2}b+h_{2}\int_{\theta T}^{b}G(t)dt \end{array}$$ (19)

$$\begin{array}{l} \frac{\partial C^{c}}{\partial Q}=e(1-G(\theta T))+h_{2}T(1-G(\theta T))+s_{2}(F(I+Q-yT)-1)G(\theta T)\\ \;+h_{2}\frac{Q}{y}G(\theta T)+eF(I+Q-yT)G(\theta T)+\frac{h_{2}}{y}G(\theta T)\int_{I+Q-yT}^{I}F(x)dx \end{array}$$ (20)

Since $\frac{\partial^{2}C^{c}}{\partial I^{2}}\geq 0$ and $\frac{\partial^{2}C^{c}}{\partial Q^{2}}\geq 0$, the optimal solutions of *I* and *Q* can be derived from Eqs (19) and (20). However, from these two expressions, we find that Eq (20) is same as Eq (15).

**Theorem 2**. In this centralized setting, the optimal ordering policies of the ERC and the hospital are:

1. The optimal ordering policy for the hospital $Q^{c*}$ would be same as that in the decentralized setting (Theorem 1) when $I^{c*}=a$ or $I^{c*}=b$.
2. Let $\nabla =\frac{s_{1}G(\theta T)-r(1-G(\theta T))-h_{1}T-e(1-G(\theta T))-h_{2}T+h_{2}b-h_{2}\int_{\theta T}^{b}G(t)dt}{s_{1}G(\theta T)+rG(\theta T)}$, and if $I^{c*}=F^{-1}(\nabla )$, then $Q^{c*}=yT$ when Ψ(*F*^−1^(∇)) ≤ 0, $Q^{c*}=\theta yT$ when Φ(*F*^−1^(∇)) ≥ 0, and when Φ(*F*^−1^(∇)) ≤ 0 together with Ψ(*F*^−1^(∇)) ≤ 0, $Q^{c*}$ can be derived by $\begin{array}{l} s_{2}(1-F(F^{-1}(\nabla )+Q^{c*}-yT))G(\theta T)=h_{2}T(1-G(\theta T))+h_{2}Q^{c*}/yG(\theta T)\\ +h_{2}/y\;G(\theta T)\int_{F^{-1}(\nabla )+Q^{c*}-yT}^{F^{-1}(\nabla )}F(x)dx+e(1-G(\theta T))+eF(F^{-1}(\nabla )+Q^{c*}-yT)G(\theta T) \end{array}$.

The proof of Theorem 2 is straightforward when we let Eqs (19) and (20) equal zero. Note that the first statement illustrates that it may not be beneficial to design certain coordination contracts to make ordering decisions in the centralized setting, which is counter intuitive. Rather, the designed contracts may incur extra costs, and the total costs in the centralized setting are not less than the total costs as a whole in the decentralized setting, which results in the extra costs outweighing the benefit of the designed contracts.

**Proposition 3**. When the optimal ordering policy for the ERC in the centralized setting is I* = *F*^−1^(∇), and I* = *F*^−1^(Δ) in the decentralized setting, we have:

1. If $-e(1-G(\theta T))-h_{2}T+h_{2}b-h_{2}\int_{\theta T}^{b}G(t)dt\geq (v-k\theta T)+k\int_{a}^{\theta T}G(t)dt$, then ∇ ≥ Δ.
2. If $\nabla ((v-k\theta T)+k\int_{a}^{\theta T}G(t)dt))>-e(1-G(\theta T))-h_{2}(T-b+\int_{\theta T}^{b}G(t)dt)$, then ∇ ≥ Δ.

To find the difference between centralized and decentralized settings, we compare ∇ with Δ, and solve for the equilibrium solutions of the ERC in centralized and decentralized settings separately. Note that 0 < ∇ < 1 and 0 < Δ < 1 mean that $-(s_{1}+r)G(\theta T)<-r-h_{1}T-e(1-G(\theta T))+h_{2}(b-T-\int_{\theta T}^{b}G(t)dt)<0$ together with $-(s_{1}+r)G(\theta T)<-r-h_{1}T+(v-k\theta T)(1-G(\theta T))<-(v-k\theta T)G(\theta T)-k\int_{a}^{\theta T}G(t)dt$. Being confined to these conditions, the value interval of parameter *θ* must be restricted. The condition $-e(1-G(\theta T))+h_{2}(b-T-\int_{\theta T}^{b}G(t)dt)<(v-k\theta T)(1-G(\theta T))$ guarantees that the marginal benefit is larger than the marginal costs of expiration and inventory in the hospital, so the optimal stock level in the decentralized setting is higher than that in the centralized setting.

Since ∂*Q*/∂*I* < 0 given the implicit function derivation, a lower stock level at the ERC means a larger ordering quantity for the hospital in the centralized setting, which is intuitive. Therefore, the total ordering quantity for emergency medicines from the supplier in this centralized setting may decrease for two different reasons: (1) the ERC’s inventory is high enough to lessen the hospital’s ordering quantity; and (2) higher costs of expiration and inventory management.

## 6. Numerical cases study

We apply a numerical case study to our proposed closed-loop emergency medicine supply chain, aiming to intuitively present the optimal ordering policies for the ERC and the hospital in the decentralized and centralized settings. Both scenarios are considered in this case study, and we find some interesting conclusions.

**Fig 9:**
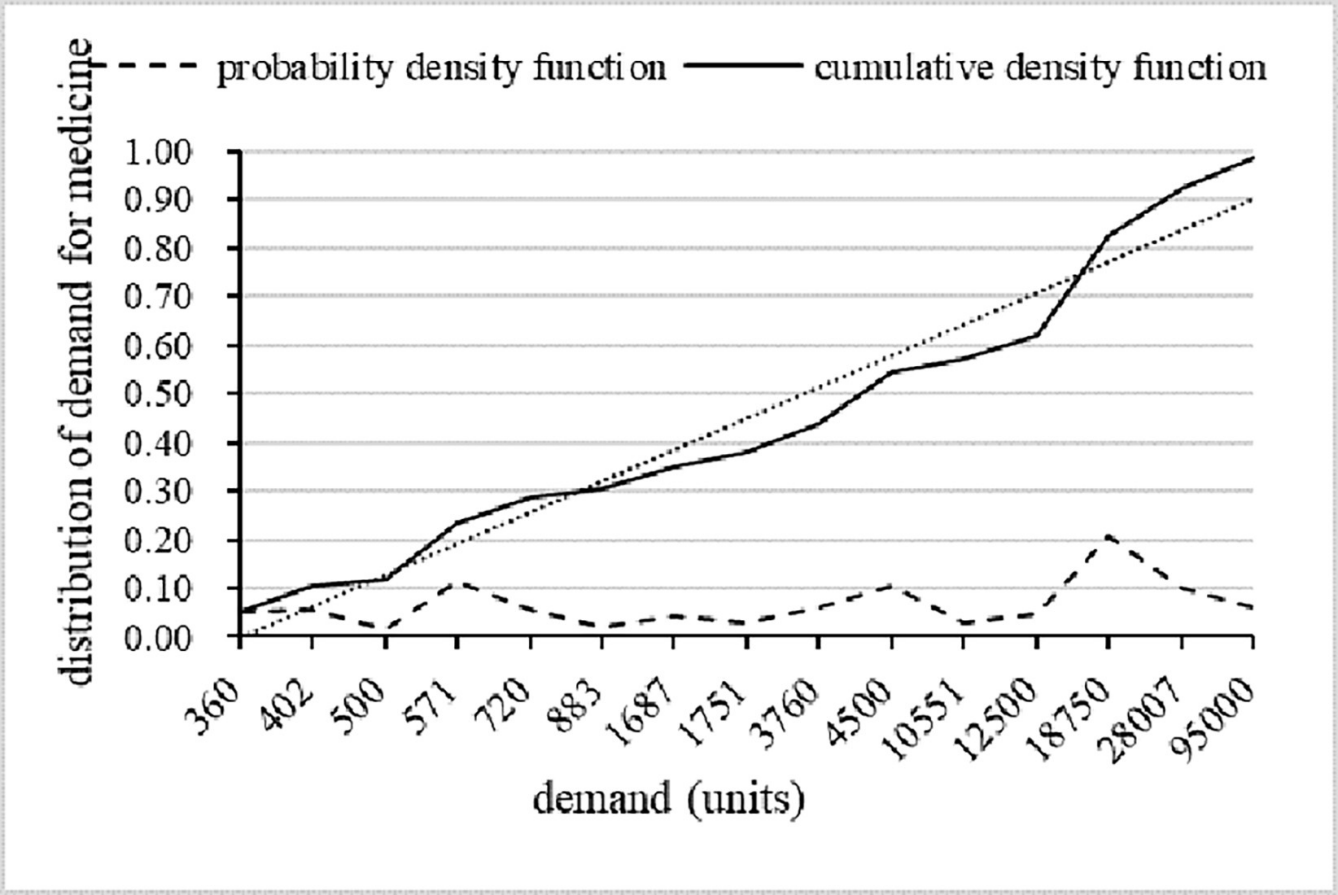
The density functions for demand in disasters.

This numerical case study is conducted as an experiment focusing on earthquake disasters in China using our proposed model. The disaster data is taken from the earthquake website [26] for the period from 1999–2017; there were 8 large-scale disasters (larger than M7) in these 18 years. In addition to this data, the occurrence time can be estimated by a uniformly distributed function, and the probability density function is *g*(*t*) = 0.036. The demand data is estimated according to the data provided by Rawls and Turnquist (2011) [27], and the cumulative density function of the stochastic demand is *F*(*x*) = (*x* – 360)/(9500 – 360) with an interval of [360,95000], as presented in Fig 9. Other parameters are estimated as the work of Mete and Zabinsky (2010) [28] suggested. The initial value of emergency medicine is *v* = 140 dollars, and the shelf life of the emergency medicine is 12 months; thus, we can derive a value of *k* = 11.67 dollars/month (140/12). Based on the work of Sheu (2007) [29] and the different prices in urban and rural areas, the holding cost in the ERC is 4 dollars/unit/month and 6 dollars/unit/month in the hospital, and the transportation cost is 15 dollars/unit. In this paper, the penalty for shortages in the ERC is 5 times that of the value of the emergency medicine; however, the shortage penalty for the hospital is less—only 4 times the value of the medicine. The first reason for this is related to the natural characteristics of emergency medicine that is used for preliminary rescues in emergency situations, and the second reason is that demand that exceeds the inventory in one hospital can be met by the transfer of inventory from other hospitals. Referring to the background from Zhou and Olsen (2017), we estimate that the demand rate for emergency medicine in China every day is 340 units—that is, 10,200 units per month. The estimated values for parameters are displayed in Table 1.

**Table 1:** The estimated values of parameters.

*V*	*T*	*k*	*r*	*s*_1_	*s*_2_	*h*_1_	*h*_2_	*e*	*y*	*g*(*t*)	*f*(*x*)
140	12	11.67	15	700	560	4	6	30	10200	0.036	1.057×10^−5^

**Fig 10:**
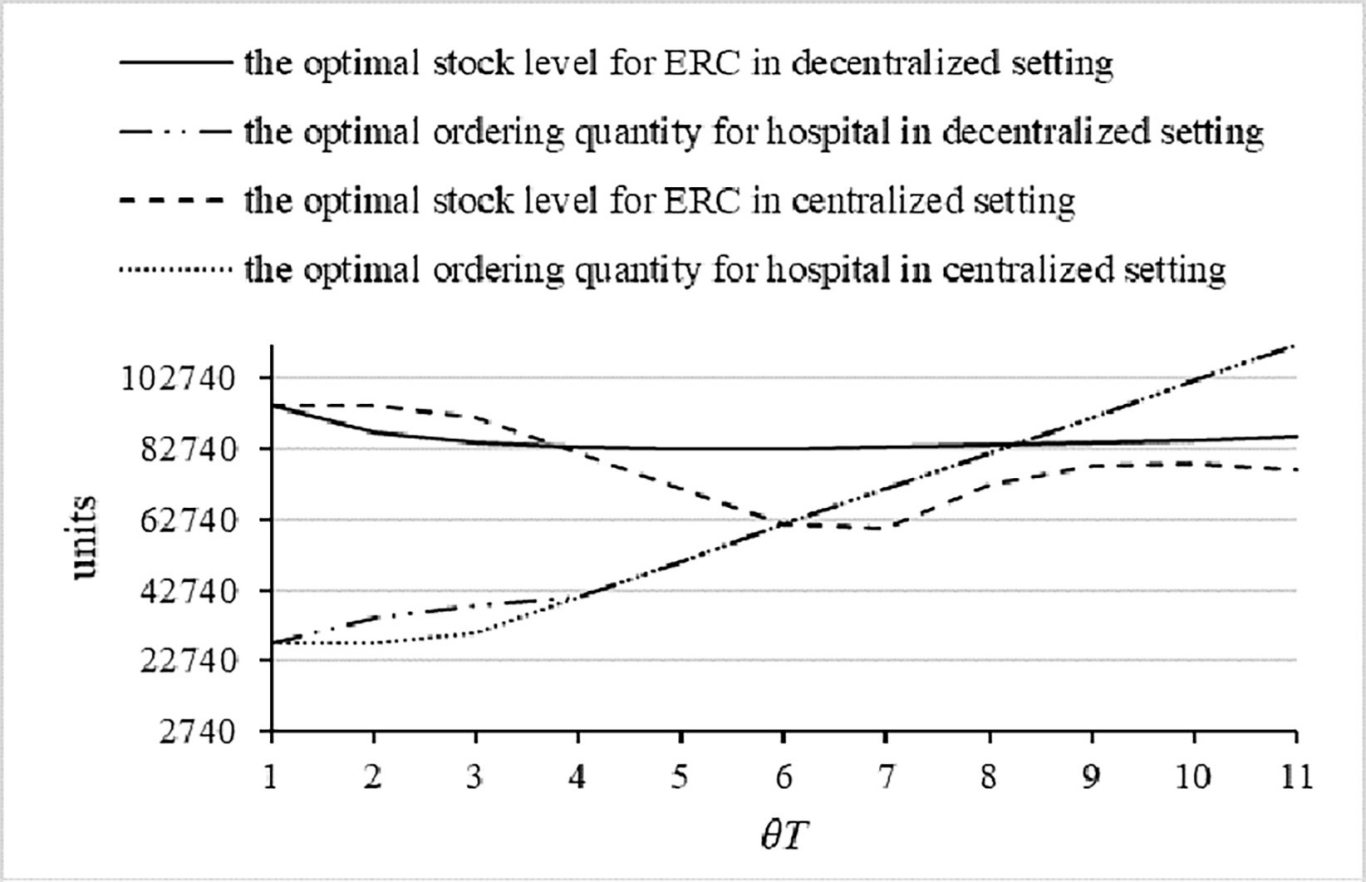
The optimal ordering policies for ERC and hospital.

First, we compute the optimal ordering policies for the ERC and the hospital and discuss how the predefined latest return time affects emergency medicine inventory decisions. Our results show a complex relationship between the predefined latest return time and the optimal ordering quantity. Several observations arise from these results, as presented in Fig 10.

One observation is that the predefined return time does matter in deciding the stock levels of emergency medicines with short a lifetime. From Fig 10, we see that the hospital’s optimal ordering quantity is up; that is to say, the later the return time, the larger the demand before that return time. However, for the ERC, the trend is first down and then up, which is counterintuitive, given that if there is a lengthy period of time before the predefined latest return time, this most likely means that the disaster was quite significant. Therefore, the possible objective reason is that there is a threshold for the designed return time. When the *θT* is smaller than the threshold—that is, the probability that a disaster will occur is relatively low—if the designed return time is lengthy, then the holding time will increase. In such a case, the ERC will decrease the inventory level to decrease holding and transportation costs. Whereas, when the *θT* exceeds the threshold—that is, the likelihood that a disaster will occur is relatively high, the ERC will maintain a higher inventory level to decrease expected shortage costs.

Our second observation is that the optimal ordering quantity for the hospital from the supplier in a centralized setting is less than in a decentralized setting, but the optimal stock level of the ERC is not the same; rather, the ERC’s inventory is first higher and then lower than it would be in a decentralized setting. From Fig 10, we can see that there is a threshold for the predefined return time of between 3 and 4 in this numerical case. When the predefined return time is earlier than the threshold, the likelihood that the reserved emergency medicines in the ERC will be used is small. In addition, the holding costs for the hospital are much higher (1.5 times) than the holding costs for the ERC. Therefore, in a centralized setting, the optimal ordering quantity of the hospital in a centralized setting is lower, and the ERC will select a higher inventory level to decrease total holding costs. However, when the predefined return time is later than the threshold value, which means that the probability that return activities will happen is low, the hospital will need to store at least as much emergency medicine as it would in a decentralized setting.

**Fig 11:**
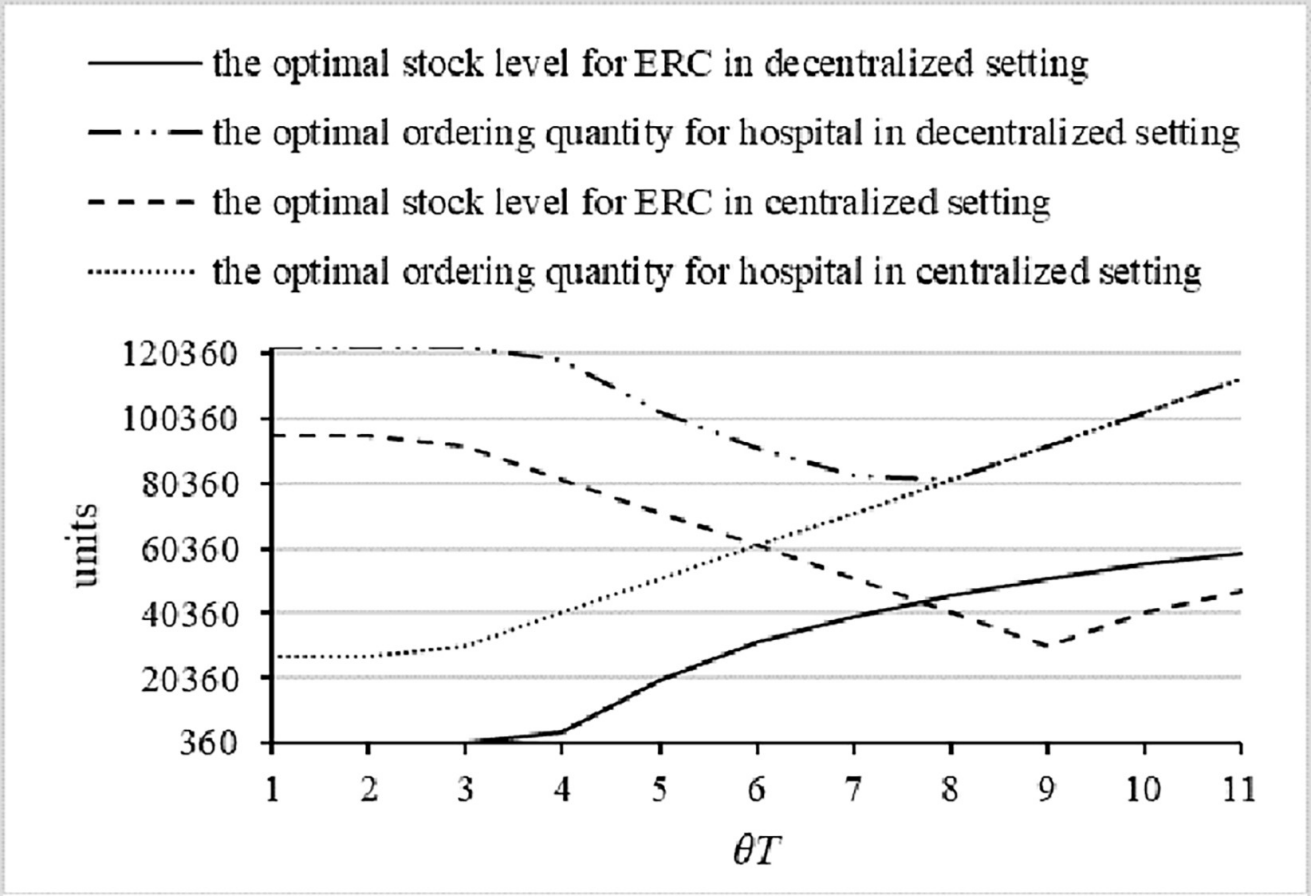
The optimal ordering policies in the case of *h*_1_ = 10 and *h*_2_ = 20.

Second, we study another case in which the expected shortage costs are less than the costs of inventory management and transfer. We conduct this case by means increased holding costs, because the shortage costs cannot be decreased easily. Comparing the results shown in Fig 11 to those shown in Fig 10, we find that the results found in the centralized and decentralized settings are similar, except that the threshold value of the predefined return time is larger. Further, the effect of the predefined return time on the optimal ordering quantity of the hospital increases, and the effect on the optimal stock level of the ERC also increases. This phenomenon can be explained as follows: the holding and transfer costs are larger, so it is viable to select the lowest stock level when the predefined return time is relatively shorter, and the optimal stock level increases with the extension of the predefined return time.

**Fig 12:**
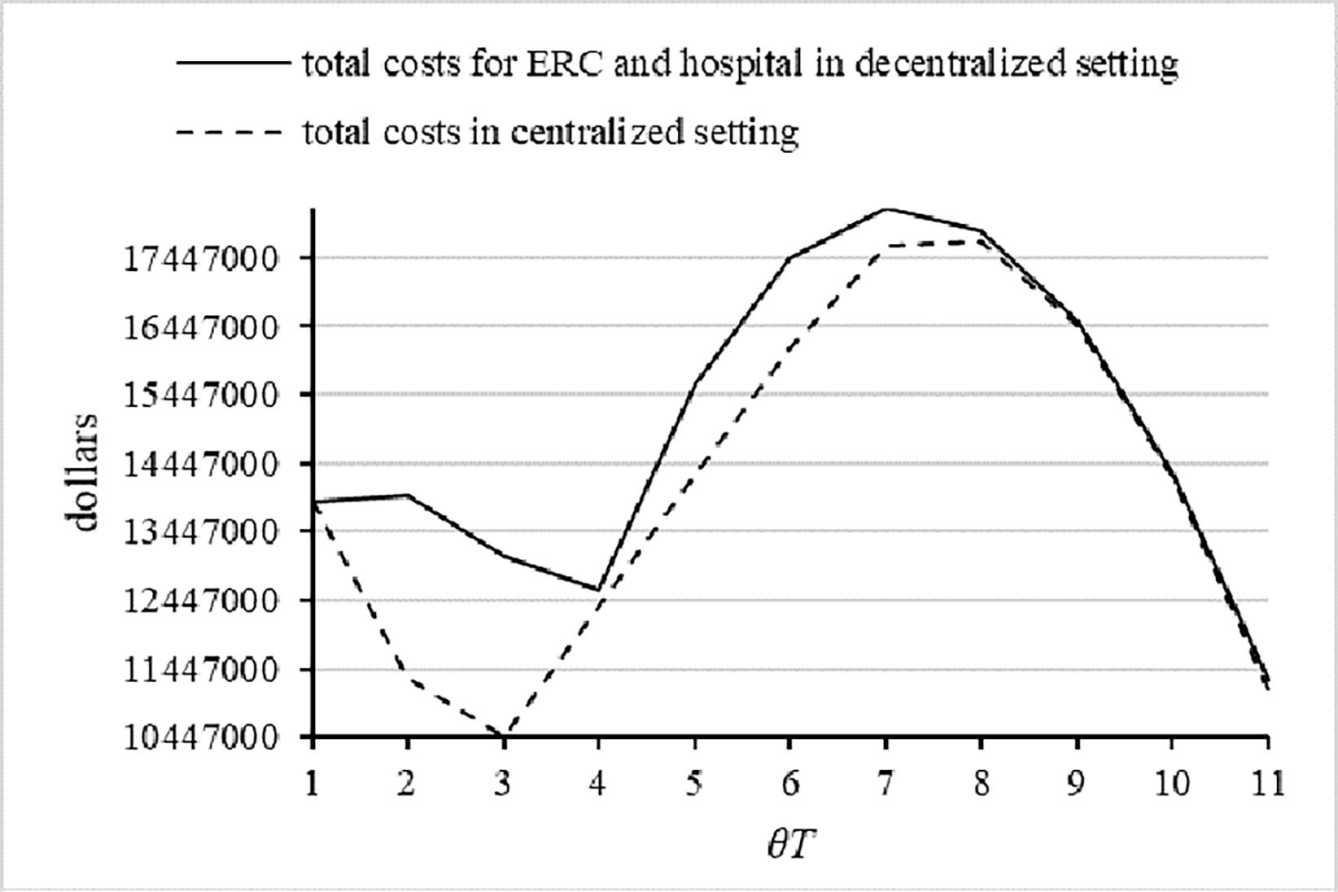
The total costs in centralized and decentralized settings.

Third, to illustrate the difference between centralized and decentralized systems, we calculate the total costs of the two systems separately, as shown in Fig 12. We obtain the same conclusion as the general research on the coordination mechanisms for the supply chain; namely, the centralized decisions perform better than the decentralized decisions. Also, when the predefined return time is brief, the difference in the two total costs increases; however, the difference size decreases when the predefined return time is relatively long. This finding can be made clear by the threshold value of the predefined return time, which is similar to the explanation of the second result in Fig 10.

**Fig 13:**
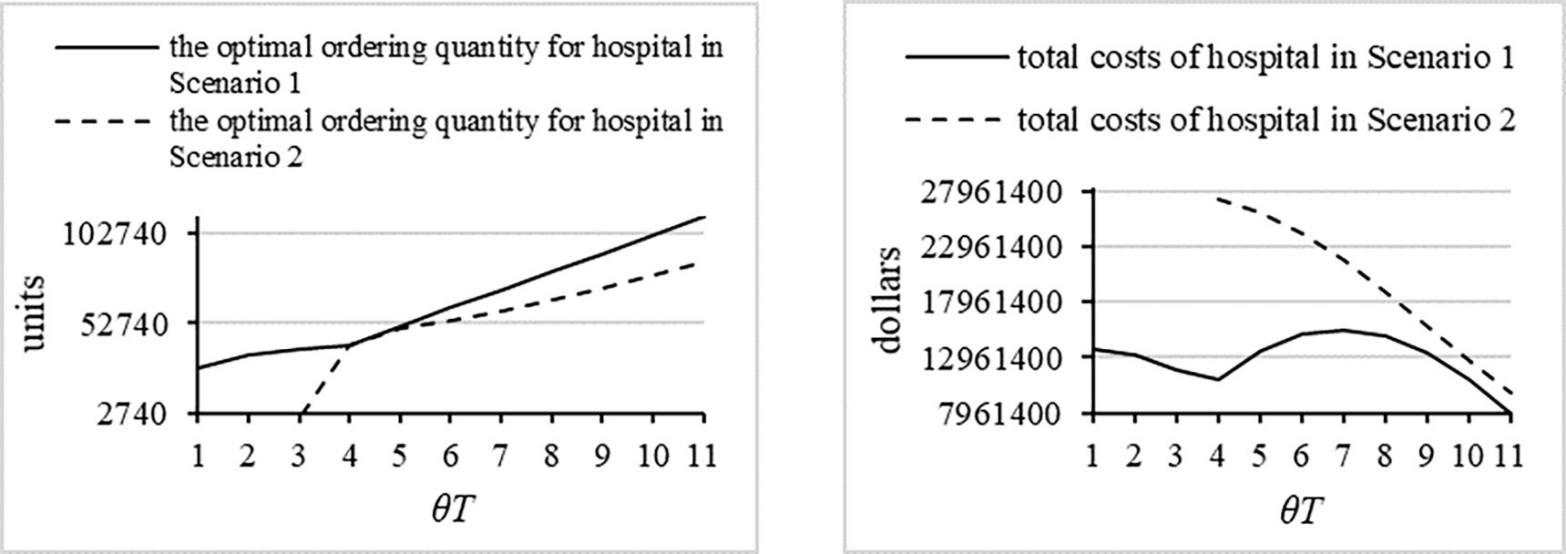
The optimal results of the hospital in Scenario 1 and Scenario 2.

Fourth, in order to intuitively reveal the optimal ordering policies in Scenario 2 in which *Q* ≤ *yθT*, we simulate the optimal ordering policies for the hospital and compute the total costs in Scenario 2. Fig 13 shows the comparisons between both scenarios. One conclusion is that the optimal ordering quantity is lower and the total costs are much higher. Further, in Scenario 2, the optimal ordering quantity increases with the increase of the predefined return time, which is same as in Scenario 1, and the increment decreases, which is different from Scenario 1. Also, in Scenario 1, the total costs of the hospital decrease with the increase in the predefined return time, because the longer the predefined return time, the lower the likelihood of a shortage in emergency medicines, which reduces the expected shortage costs. In all, the above conclusions indicate that the decisions in Scenario 2 are not viable options, so it is reasonable to omit the analysis of the results of Scenario 2.

## 7. Conclusions

In this research, we investigate a system of an emergency medicine closed-loop supply chain with an ERC, a hospital and a supplier, and derive the optimal ordering policies for the ERC and the hospital. In this study system, the return process is stochastic, since the occurrence time of a disaster and the demand for emergency medicines are uncertain. We first build models for the inventory ordering problem in a decentralized setting, and we then consider the problem in a centralized setting. We design a contract to reduce total costs by coordinating the ordering mechanisms of the ERC and the hospital. The analytical optimal policies show that the emergency medicine closed-loop supply chain is an applicable method to control emergency medicine expiration costs and waste.

By addressing the expiration problem and the ERC’s and hospital’s risk-averse behavior through an analysis of the uncertainties related to occurrence time and disaster severity, this study makes several important theoretical and practical contributions to the field of management. First, this research has furthered the application of the emergency inventory model. In a decentralized setting, models are built for all possible scenarios, which can divided into two scenarios: *Q* ≥ *yθT* and *Q* ≤ *yθT*. Scenario 2 *Q* ≤ *yθT* does perform better than Scenario 1. Intuitively, the optimal ordering quantity of the hospital from the supplier increases with the return time, but the increment first increases and then remains constant. Less intuitively, we find that the optimal stock level at the ERC first decreases and then increases as the return time lengthens; therefore, we find that there is a threshold value for the predefined return time that affects the optimal decisions differently.

Further, we find that in a centralized system, like a nationalized health system, the hospital and the ERC may be interested in cooperating with each other to reduce total costs, including expiration costs, holding costs and shortage costs. However, a challenge with implementing a centralized system is that the total amount of reserved emergency medicines at the ERC and the hospital may be lower than they would be in a decentralized system, which increases the risk that demand may exceed stock. Thus, a coordination contract should be designed to motivate the hospital and the ERC to make inventory decisions together to control the risk of shortage.

Our theoretical study has also drawn a number of practical implications that warrant further analysis. First, from our study, we find that decision-makers of ERCs need to better define the latest return time and decline appropriate values for the return cost using hard technological skills or soft training skills; for example, shifting efforts away from singular gain toward cross-sectorial collaboration to improve closed-loop supply chain performance. Therefore, to enhance emergency service levels by using a closed-loop strategy, ERC managers should try to shorten the latest return time, which requires a coordinated approach, and address positive externalities and efficiencies across the closed-loop supply chain.

An online emergency inventory system should be built that provides access to rich data sets, which can help researchers investigate decision-maker behavior related to emergency supply issues [22–25, 30]. This data would help managers better evaluate the possibility for losses in disasters or accidents; this can also help managers adopt more appropriate risk management practices and make more reliable decisions. Finally, when compared with a decentralized closed-loop strategy, the centralized closed-loop strategy provides a more reliable means of dealing with the uncertainties of occurrence time and demand. Therefore, establishing a resilient and centralized emergency supply value chain based on the remaining lifetime of products is a more efficient and productive means of managing emergency supplies.

Our work can be extended in several ways for future research. A natural extension of our study is to consider the emergency medicine closed-loop supply chain in a multi-horizon perspective, in which one shelf life horizon can be divided into multiple periods based on the occurrence time of disasters. Such an examination would provide an opportunity to develop models that evaluate the coordination mechanisms and relationships that exist between the hospital and the ERC to conduct optimal centralized policies. In addition, more complicated factors should be introduced into the emergency medicine inventory system, including investigating different product types of emergency supplies with different costs, and further exploring how demand parameters may affect optimal inventory policies.
